# Enhanced Lipid Production in *Chlamydomonas reinhardtii* by Co-culturing With *Azotobacter chroococcum*

**DOI:** 10.3389/fpls.2018.00741

**Published:** 2018-06-28

**Authors:** Lili Xu, Xianglong Cheng, Quanxi Wang

**Affiliations:** Department of Biology, College of Life and Environmental Science, Shanghai Normal University, Shanghai, China

**Keywords:** *Chlamydomonas reinhardtii*, *Azotobacter chroococcum*, co-culture, lipid production, biomass

## Abstract

The green algae, *Chlamydomonas reinhardtii*, is one of the model species used to study lipid production, although research has focused on nitrogen-deficient cultures, that inhibit the development of biomass by *C. reinhardtii* and limit lipid production. In this study, *Azotobacter chroococcum* was added to the algal culture to improve lipid accumulation and productivity of *C. reinhardtii*. The maximum lipid content and production of *C. reinhardtii* in the co-culture were 65.85% and 387.76 mg/L, respectively, which were 2.3 and 5.9 times the control's levels of 29.11% and 65.99 mg/L, respectively. The maximum lipid productivity of *C. reinhardtii* in the co-culture was 141.86 mg/(L·day), which was 19.4 times the control's levels of 7.33 mg/(L·day). These increases were attributed to the enhanced growth and biomass and the change in the activity of enzymes related to lipid regulation (ACCase, DGAT, and PDAT). Compared to the conventional strategy of nitrogen deprivation, *A. chroococcum* added to the culture of *C. reinhardtii* resulted in higher lipid accumulation and activity, greater efficiency in the conversion of proteins to lipids, higher biomass, and increased growth of *C. reinhardtii*. Therefore, using *A. chroococcum* to improve the growth and biomass of *C. reinhardtii* is an efficient, rapid, and economically viable strategy for enhancing lipid production in *C. reinhardtii*.

## Introduction

The global supply of traditional fossil fuels is limited and the combustion of fossil fuels produces CO_2_ and other greenhouse gases that cause climate change (Hui et al., 2016). There is an urgent need for new types of renewable, clean energy resources. Biodiesel is a renewable and biodegradable fuel that is considered environmentally friendly because it is produced from unprocessed or recycled vegetable oils and animal fats through various chemical reactions (Ho et al., 2014). The–supply and price of raw materials are the key limiting factors of biodiesel applications; thus, cheap and renewable raw materials for biodiesel production are required for the large-scale utilization of biodiesel.

Through photosynthesis, algae can convert CO_2_ and water to O_2_ and macromolecular organic matter in the form of carbohydrates and lipids (Hu et al., 2008; Scott et al., 2010). Under certain stress conditions, such as high light intensity or nutrient deficiency, some algae can accumulate large amounts of lipids, such as triacylglycerides. Because of their fast growth, high lipid content, and optimized lipid composition, microalgae are ideal materials for biodiesel production (Hu et al., 2008; Wang et al., 2009; Siaut et al., 2011). *Chlamydomonas reinhardtii* (*C. reinhardtii*) is a unicellular green algae species, whose genome has been fully sequenced. It grows quickly, costs little to cultivate, and can produce lipids under nitrogen-deficient conditions; thus, it has been widely used for lipid production (Park et al., 2015). As with other microalgae, the growth of *C. reinhardtii* is repressed by nitrogen deficiency. Algal biomass and lipid accumulation showed a negative correlation with nitrogen deficiency (Park et al., 2013, 2015; Fan et al., 2014). This has resulted in lower lipid accumulation and productivity than its theoretical capacity. The ideal model involves increasing lipid accumulation using green algae by removing nitrogen from the medium without limiting the algal biomass. In order to improve the lipid accumulation by *C. reinhardtii*, we need to identify an effective way to increase the biomass of *C. reinhardtii* in the absence of nitrogen.

In natural environments, bacteria and algae share a complex ecological relationship. Some bacteria can promote the growth and biomass of algae by metabolic complementarity. Ietswaart et al. (1994) found that two obligate aerobic bacteria, *Pseudomonas diminuta* and *P. vesicularis*, could promote the growth of *Scnedesmus obliquus* and *Chlorella* sp. Bell et al. (1974) reported that *Skeletonema costatum* cultivated alongside *Pseudomonas* strain grew faster than in its absence and did not survive without *Pseudomonas*. Riquelm et al. (1988) suggested that the glycoprotein secreted by *Pseudomonas* promoted the growth of *A. glacialis*. Some marine microalgae and heterotrophic bacteria, when co-grown, can secrete extracellular enzymes or specific growth factors that promote each other's growth. These include *Bacillus halmapalus* and *Alexandrium tamarense* (Zheng et al., 2002). *Azotobacter chroococcum* (*A. chroococcum*) is a nitrogen-fixing aerobic bacteria species that can draw nitrogen from the air (Walker and Yater, 1978). It is widely used in the study of biochemical processes, electron transport, and iron storage (Krakow and Ochoa, 1963). Great progress has been made in understanding the biochemistry and genetics of hydrogen metabolism and nitrogen fixation by studying *A. chroococcum*. These studies determined the ability of *A. chroococcum* to stimulate plant growth through the production of plant growth substances and fixed nitrogen, and other factors (Rubenchik, 1963).

In this work, to increase the biomass and lipid accumulation of *C. reinhardtii*, we co-cultured *C. reinhardtii* cc849 with *A. chroococcum* under nitrogen-deficient conditions and investigated the underlying mechanism of the resulting increased lipid content. The lipid content and lipid productivity of *C. reinhardtii*, when mixed with *A. chroococcum* were monitored. The growth, biomass, cellular biochemical components and fatty acid of *C. reinhardtii* in the co-culture and pure algal culture were compared in this study. Finally, the transcription levels of the genes related to the lipid production of algae and its co-culture with *A. chroococcum* were analyzed. The special ecological relationship between green algae and bacteria was used to improve the biomass and lipid content of *C. reinhardtii*, which is a clean and sustainable way to produce biofuel. This research provides a novel and useful strategy to enhance the lipid accumulation and productivity of *C. reinhardtii* for treatment by the co-culture with bacteria.

## Materials and methods

### Algal and bacterial strains and culture conditions

*Chlamydomonas reinhardtii* cc849 was purchased from the *Chlamy* Center. It is a type of cell deficient strain. The algae grew in the Tris-acetate-phosphate (TAP) medium (pH = 7.0) under light conditions of 100 μE·m^−2^·s^−1^ at 25 ± 1°C (Harris, 2009). The cell density was determined by absorbance at 750 nm (OD_750_). Chlorophyll of *C. reinhardtii* was extracted with alcohol. Cells (1 mL) were extracted and centrifuged at 12,000 g for 1 min at room temperature and the supernatant was removed. Then, 1 mL of 95% alcohol was added to the tube and the pellet was resuspended and centrifuged at 12,000 g for 1 min at room temperature (Harris, 2009). The supernatant was extracted and absorbance was measured at 665 nm and 649 nm (Wu et al., 2011).

$$\begin{array}{l} \text{Chlorophyll content }\left(\text{mg}/\text{L}\right)\;=\text{ O}{\text{D}}_{665}\;\times \;6.01+\text{O}{\text{D}}_{649}\;\times \;20.04 \end{array}$$

*Azotobacter chroococcum* No 1.0233 was purchased from China General Microbiological Culture Collection Center (CGMCC) and cultured in a nitrogen fixation medium (pH = 7.0) at 28 ± 1°C. The cell density is expressed as the absorbance at 600 nm (OD _600_) (Winogradski, 1935). The cell density of *A. chroococcum* in the co-culture was obtained by OD_600_ of the mixture minus the OD_600_ of pure algal culture.

$$\begin{array}{lll} {\text{OD}}_{600\;\left(\text{bacteria in co}-\text{culture}\right)}\; & = & \;{\text{OD}}_{600\;\left(\text{co-culture}\right)}\\ -\;{\text{OD}}_{600\;\left(\text{pure algal culture}\right).} \end{array}$$

### Co-culture of algae and bacteria for lipid production

The algal cells in the culture flask were harvested by centrifugation at 4,500 g and 25°C for 5 min when they grew to saturation. The algal pellet was washed gently with the TAP-N medium three times to remove nitrogen thoroughly (Wan et al., 2013). The bacterial medium was also harvested by centrifugation at 5,000 g and 25°C for 5 min when it is grown to saturation then resuspended with the TAP-N medium three times. The bacterial pellet was resuspended with a suitable volume of the TAP-N medium so that OD_600_ = 1.0. Then, 10 mL of the resuspended bacterial sample was added to a 500 mL flask followed by the addition of 0.5 mg algae. Finally, the TAP-N medium was replenished in the flask. The sample in the flask was shaken gently so that bacteria and algae are mixed well. The flask was placed under 200 μE·m^−2^·s^−1^.

Samples were dried in an oven at 80°C for 24 h until the weight ceased to decrease (dry weight is designated as DW here). Ten microliter of the bacterial culture broth was mixed with 990 μl water and spreaded on a solid plate medium, then the number of colonies were counted after culturing in 28°C for 48 h. The cells number/mL of algae was obtain through the microscope observation. The linear relationship between cells number/mL and biomass of *C. reinhardtii* and *A. chroococcum* were determined. The cells number/mL of algae and bacteria in the co-culture could obtain by microscopy and plate medium coating, respectively. The biomass of bacteria and algae in the co-culture medium were obtained by the following equation:

$$\begin{array}{lll} {\text{DW}}_{\text{bacteria}} & = & \text{Cell number}/\text{mL }\times \;0.0557\;+\;0.032\;{\text{R}}^{2}\;=\;0.9931;\\ {\text{DW}}_{\text{algae}}\; & = & \text{ Cell number}/\text{mL }\times \;0.8451\;+\;0.263\;{\text{R}}^{2}\;=\;0.9923. \end{array}$$

### Observation of *C. reinhardtii* and *A. chroococcum* by microscopy

In this experiment, 1 mL of the cells of the culture was stained with Nile Red (Sigma) by adding the dye to a final concentration of 1 mg/mL and running the reaction for 15 min. The bright-field images of *C. reinhardtii* cells grown in the TAP-N media were captured using a confocal laser scanning microscope (Nikon Eclipse 80i) equipped with a digital camera (Nikon DS-Ri1), respectively. Subsequently, the corresponding fluorescence images of the Nile Red signal were captured by the excitation line at 488 nm (Chen et al., 2009; Boyle et al., 2012).

### Extraction and detection of lipid content

Total lipids were extracted using a modified version of the protocol reported by Bligh and Dyer (1959). Here, 400 mL of algal cells were harvested by centrifuging at 7,500 g for 10 min and the sediment was washed with fresh TAP-N medium three times. Solid samples were placed in dry weighing bottles and dried in an oven at 80°C for 24 h until the weight ceased to decrease. Then, 0.2 g (weight is designated W_0_) of dry cells were transferred into the centrifugal tube. Subsequently, 5 mL mixture of chloroform and methyl alcohol at a volume ratio of 1:1 were added to the centrifugal tube. The cells in the centrifugal tube were shaken for 30 min, placed in a centrifuge tube, and centrifuged at 8,000 g for 10 min. All the steps were repeated until the supernatant was colorless. The supernatant was collected (weight is designated W_1_) and transferred, to a dry rotary evaporator, of known weight, and evaporated to dryness (weight was designated W_2_).

Biomass (mg) = DW;

Unit biomass (mg/L) = DW/0.5;

Lipid content = (W_2_ – W_1_)/ W_0_ × 100%;

Lipid production (mg/L) = W_2_;

Lipid productivity (mg/(L·day)) = W_2_/day.

### Fatty acid analysis of lipid of algae and *A. chroococcum*

Fatty acids in algae were analyzed using a previously described method (Wang et al., 2013) with some modifications. First, 0.1 g dry sample was added to a 15 mL glass vial and then it was dissolved in a 2 mL methanolic HCl and 3 mL chloroform-methanol solution (volume ratio 1:1). Finally, 1 mL heptane containing 50 μg heptadecanoic acid methyl ester (C_18_H_37_-COOCH_3_) as the internal standard was added to the glass vial. The reaction proceeded at 85°C for 1 h, after which 1 mL hexane was added to the vial. The solution was left to stand for 1 h to obtain the supernatant, which was used for FAME analysis. Samples were detected by GC-MS (Thermo, Fisher, ISQ) equipped with a flame ionization detector (FID) and Agilent HP-5 GC Capillary Column (30 m × 0.25 mm × 0.25 μm). Nitrogen was used as a carrier gas. The injector temperature was set at 290°C with an injection volume of 2 μL under split mode (10:1). The detector temperature was set at 280°C. The individual FAMEs were identified by comparing their retention time with those of the authentic standards.

### Determination of protein content and carbohydrate content

The protein content of *C. reinhardtii* was measured using the BCA method (Hu et al., 2008). Algal cells (5 mL) were harvested and centrifuged at 7,500 g for 10 min. The supernatant was discarded. Subsequently, 1 mL of 15 mM KH_2_PO_4_ (pH 4.5) and 2 mL of 20% NaOH were added to the tube and shaken for 30 s. The tube was put into boiling water for 10 min, and then centrifuged at 7,500 g for 10 min. The supernatant was collected and used to assay the protein content using Pierce BCA protein assay kit (Thermo). Bovine serum albumin was used as the standard sample to obtain a standard curve. Subsequently, the protein content was calculated from the absorbance measured using the microplate reader at 560 nm and the standard curve. Carbohydrate content of *C. reinhardtii* was detected by anthrone colorimetry (Dubois et al., 1956). First, 10 mg of algal powder was put into a tube and synchronously, 0.5 N H_2_SO_4_ was added. Then the tube was incubated in a bath at 80°C for 1 h. Finally, the reactant was centrifuged at 8,000 g for 5 min to collect the supernatant and the steps were repeated once. The supernatant was extracted and the absorbance was measured at 625 nm (Dubois et al., 1956).

### RNA extraction and real-time quantitative PCR

Two milliliters of cells were collected from the samples on days 0, 1, 5, and 9 in the TAP-N medium and RNA was extracted using a QIAGEN Plant Mini Kit. The concentration and purity of the extracted RNA were measured using a UV spectrophotometer. Single stranded cDNA was synthesized from 2 μg of DNA-digested total RNA following the reverse transcription protocol provided by the manufacturer (Promega™). Transcriptional levels of genes related to lipid accumulation were detected using real-time quantitative PCR. Primers for quantitative real-time RT-PCR were designed using the Primer 5 software and are shown in Table 1. Real-time quantitative PCR was performed as stipulated by the manufacturer of the SYBR Green real-time PCR Master Mix Kit (TOYOBO™, Japan). The actin gene from *C. reinhardtii* was used as an internal control to normalize the differences between the loading amounts of the template (Makarova et al., 2007). Each PCR reaction contained 1 μL (8 ng) of cDNA, 10 μL of SYBR Green 2 × Master Mix, and 1 μL of each gene-specific primer pair (10 mM) to a final volume of 20 μL. PCR was performed as follows: 95°C for 10 min followed by 40 cycles at 95°C for 10 s, 60°C for 1 min, and 72°C for 30 s. PCR products were analyzed using the Dissociation Curves Software of ABI. The 2^−ΔΔct^ method was used to calculate the fold changes of differentially expressed genes.

**Table 1:** Primers used for real-time quantitative PCR.

**Gene Name**	**Accesion Number**	**Primer**
*ACTIN*	XM_001699016	F′-ATGGGCCAGAAGGACTCGTA
		B′-GTCGTCCCAGTTGGTCACAA
*ACC*	XM_001703135	F′-CAAGACTCTGGTTAGCGATGC
		B′-CCCAAAGCGAGACAGGATAG
*DGAT1*	XM_001693137	F′-ACTGGTGGAATGCGGCTAC
		B′-TAGCAGCTCGTGGAACACAG
*DGTT1*	KC788199.1	F′-CGGCGGAGGGAACTTAT
		B′-GAAGAGGTGCGGGGACA
*DGTT2*	KC788200.1	F′-GTTCCCGCACGGTGTCTT
		B′-ACTTCGTTCCTTCGCACC
*DGTT3*	KC788201.1	F′-GTCAGAGCCAAGTGCTGGAC
		B′- TCCACCTCCTTGTCGAACTC
*DGTT4*	KC788202.1	F′-TGCCAGATGGAAGGTGGAGTG
		B′-GTAAGCATGTGCGGTGAAGGG
*DGTT5*	XM_001701615.1	F′-GCCGTCACAGGGCTTGGGAGAA
		B′-TCCGCCTGTGCCTCTGACGG
*PDAT1*	AFB 73928	F′-AGCACAAAGCCGTGTCGATG
		B′-TTGCCCAGGATGTCGATGTG
*PEPC2*	XM_001695765	F′-CGTGAACCCCCGTAGAAAAG
		B′-CGGAGACAGTCGTCAAGCAG

### Statistical analysis

All experiments were repeated three times independently, and data were recorded as the mean with SD. Statistical analyses were conducted using SPSS 19.0. Spearman correlation coefficients were computed. The results with a *P*-value < 0.05 was considered statistically significant. “^*^” indicates a significant differences between the experimental and control groups.

## Results and discussion

After *A. chroococcum* was added to the algal culture, the maximum lipid production of *C. reinhardtii* in the co-culture was 387.76 mg/L, which was 65.85% of the composition of the algal cell. The maximum lipid productivity of *C. reinhardtii in* the co-culture was 141.86 mg/(L·day), which was 19.4 times the control's value of 7.33 mg/(L·day). These were attributed to enhanced growth and biomass. Furthermore, compared with the controls, the activity of enzymes related to lipid regulation, ACCase, DGAT, and PDAT was changed.

### Growth and biomass of algae co-cultured with *A. chroococcum*

**Figure 1:**
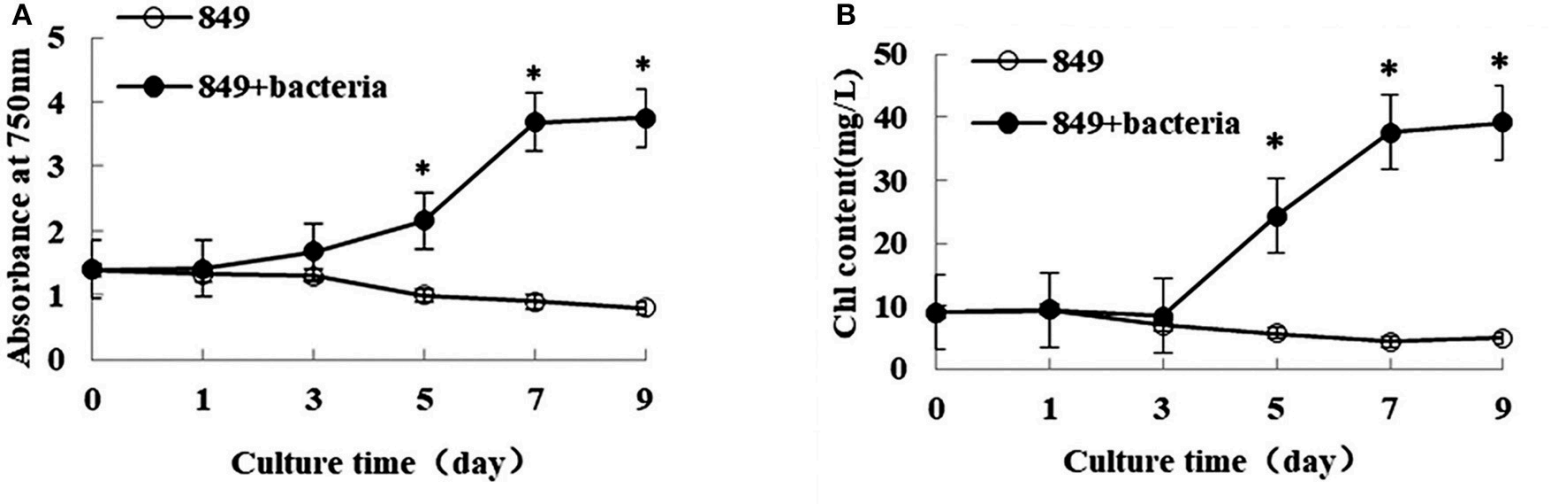
OD_750_ **(A)** and chlorophyll content **(B)** of algae in the co-culture were measured on days 0, 1, 3, 5, 7, and 9 after incubation in a nitrogen-deprived medium. Pure algae culture was used as the control. Light intensity was 200 μE·m^−2^·s^−1^ and the volume ratio of bacteria (OD_600_ = 1.0) and (OD_750_ = 1.0) was 1:40. ^*^significant difference between the experimental and control groups. The vertical bars indicate standard errors calculated from at least three independent experiments.

Lipid production was directly related to algal biomass; thus, the growth and biomass of algae and the algae co-cultured with *A. chroococcum* were detected under nitrogen deprived conditions. The initial OD_750_ value of both pure algae and the algae co-cultured with *A. chroococcum* was 1.42. After nitrogen deprivation, OD_750_ of pure algae gradually decreased and reached a minimum value of 0.91, while OD_750_ of algae co-cultured with *A. chroococcum* increased sharply, especially after day 3. It peaked at 3.75, which was 4.1 times the control value, on day 9 (Figure 1A). Consistent with the change in OD_750_, the initial chlorophyll content of both pure algae and algae co-cultured with *A. chroococcum* was 9.95 mg/mL. After nitrogen starvation chlorophyll content of pure algae decreased slightly and reached the minimum value of 4.85 mg/mL on day 9. Conversely, the chlorophyll content of algae co-cultured with *A. chroococcum* increased significantly after day 3. It peaked at 39.13 mg/mL, which was 8.1 times that of pure algae on day 9 (Figure 1B). A significant difference in the growth of algae co-cultured with *A. chroococcum* and pure algal culture was detected by *t-test* on days 3, 5, 7, and 9 (*p* < 0.05).

**Figure 2:**
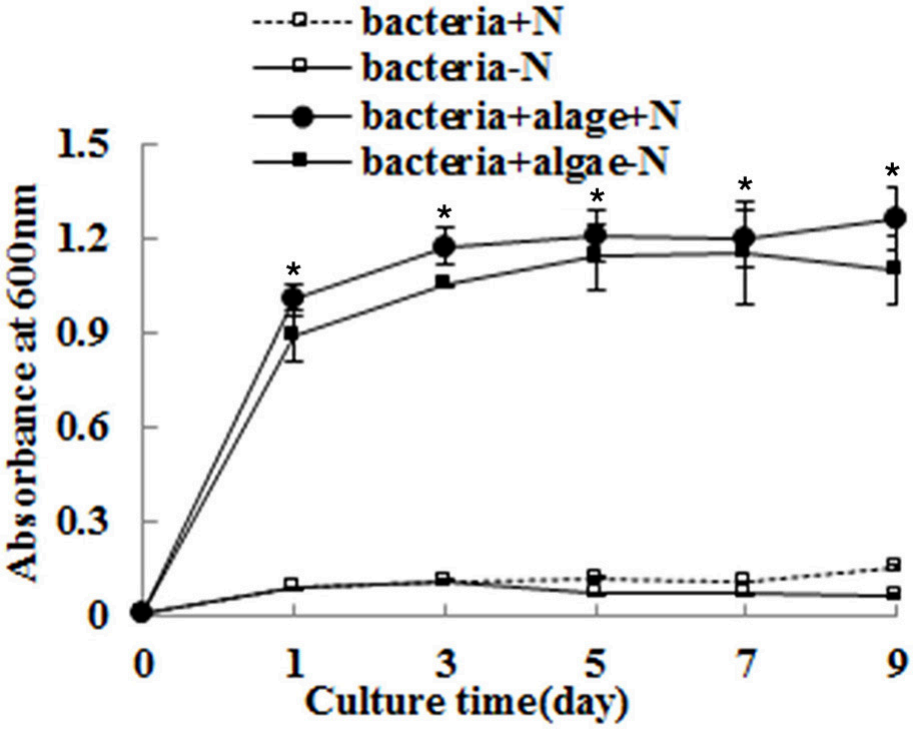
The growth of *A. chroococcum* co-cultured with algae were measured in TAP and TAP-N media. Pure bacterial culture in both media was used as controls. Light intensity was 200 μE·m^−2^·s^−1^. The same condition as the algae in the co-culture produced lipids. The volume ratio of bacteria (OD_600_ = 1.0) and algae (OD_750_ = 1.0) was 1:40. ^*^significant difference between the experimental and control groups. The vertical bars indicate standard errors calculated from at least three independent experiments.

The growth of *A. chroococcum* co-cultured with algae in TAP-N was monitored and the results are shown in Figure 2. Pure algae cells in TAP and TAP-N media were used as controls. The results indicated that the initial OD_600_ value of both pure *A. chroococcum* and *A. chroococcum* co-cultured with algae was 0.006. The OD_600_ value of pure *A. chroococcum* in both media increased slightly from day 1 to day 9 and reached the maximum value of 0.15 and 0.07 on day 9, respectively. The OD_600_ value of *A. chroococcum* co-cultured with algae in both media increased significantly and reached the maximum value of 1.27 and 1.16 on day 9 and day 7, respectively. A significant difference between the growth of *A. chroococcum* co-cultured with algae and pure algae in the TAP and TAP-N media was detected by *t-test* on days 1, 3, 5, 7, and 9 (*p* < 0.05).

**Figure 3:**
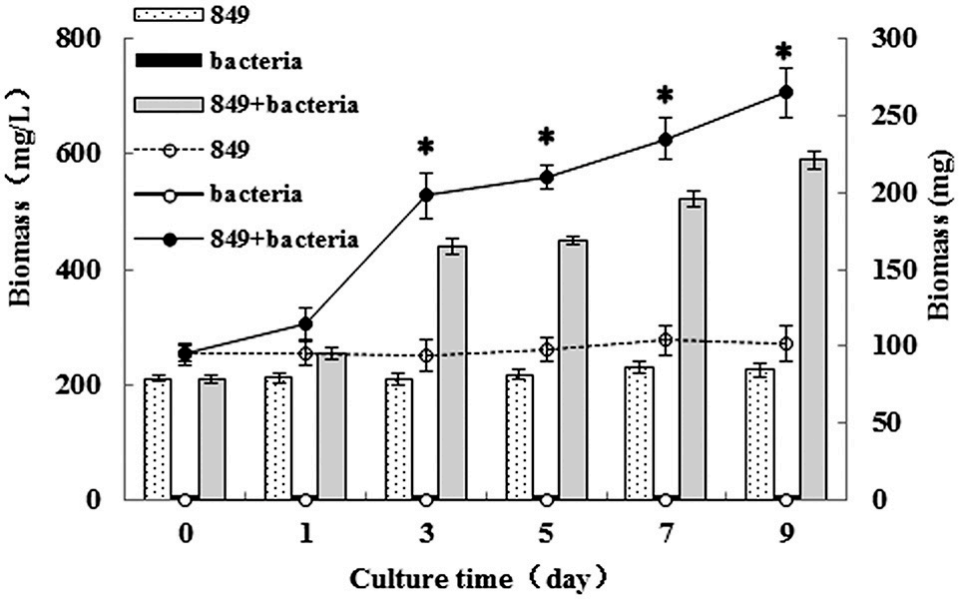
Unit biomass (mg/L) and total biomass (mg) of algae in the co-culture were measured on days 0, 1, 3, 5, 7, and 9 after incubation in a nitrogen-deprived medium. Pure algae culture was used as the controls. Unit biomass is shown with bars and total biomass is shown with dashed and solid lines, respectively. ^*^significant difference between the experimental and control groups. The vertical bars indicate standard errors calculated from at least three independent experiments.

The biomass of algae co-cultured with *A. chroococcum* was also monitored and the results are shown in Figure 3. Pure algae and bacteria were used as controls. Biomass of pure algae and pure bacteria was labeled by the dry weight (DW) of algal and bacterial cells. Biomass of algae co-cultured with *A. chroococcum* was calculated by reducing the weight of the pure bacteria cells (Figure 3). The results indicated that nitrogen deprivation caused a decline in the biomass of pure algae. However, the biomass of algae co-cultured with *A. chroococcum* increased (Figure 3). The total biomass of the pure algae decreased from 95.00 mg to the minimum value of 90.01 mg on day 9 (Figure 3). Total biomass of the algae mixed with *A. chroococcum* dramatically increased and reached the maximum value of 265 mg, 2.9 times the control. As with the change in total biomass, the initial unit biomass of pure algae and samples mixed with *A. chroococcum* was 211 mg. The unit biomass of pure algae decreased slightly to the minimum value of 200.00 mg on day 9, while the total biomass of algae mixed with *A. chroococcum* increased to the maximum value of 588 mg, also 2.9 times the control. A significant difference between the biomass of algae co-cultured with *A. chroococcum* and pure algal culture was observed by *t-test* on 3, 5, 7, and 9 days (*p* < 0.05). The number of bacteria added to the algal culture was mostly small. Total biomass and unit biomass of pure *A. chroococcum* slightly increased from the initial value of 0.006 mg and 0.015 mg/L to the maximum value of 0.010 mg and 0.025 mg/L, respectively (Figure 3).

**Figure 4:**
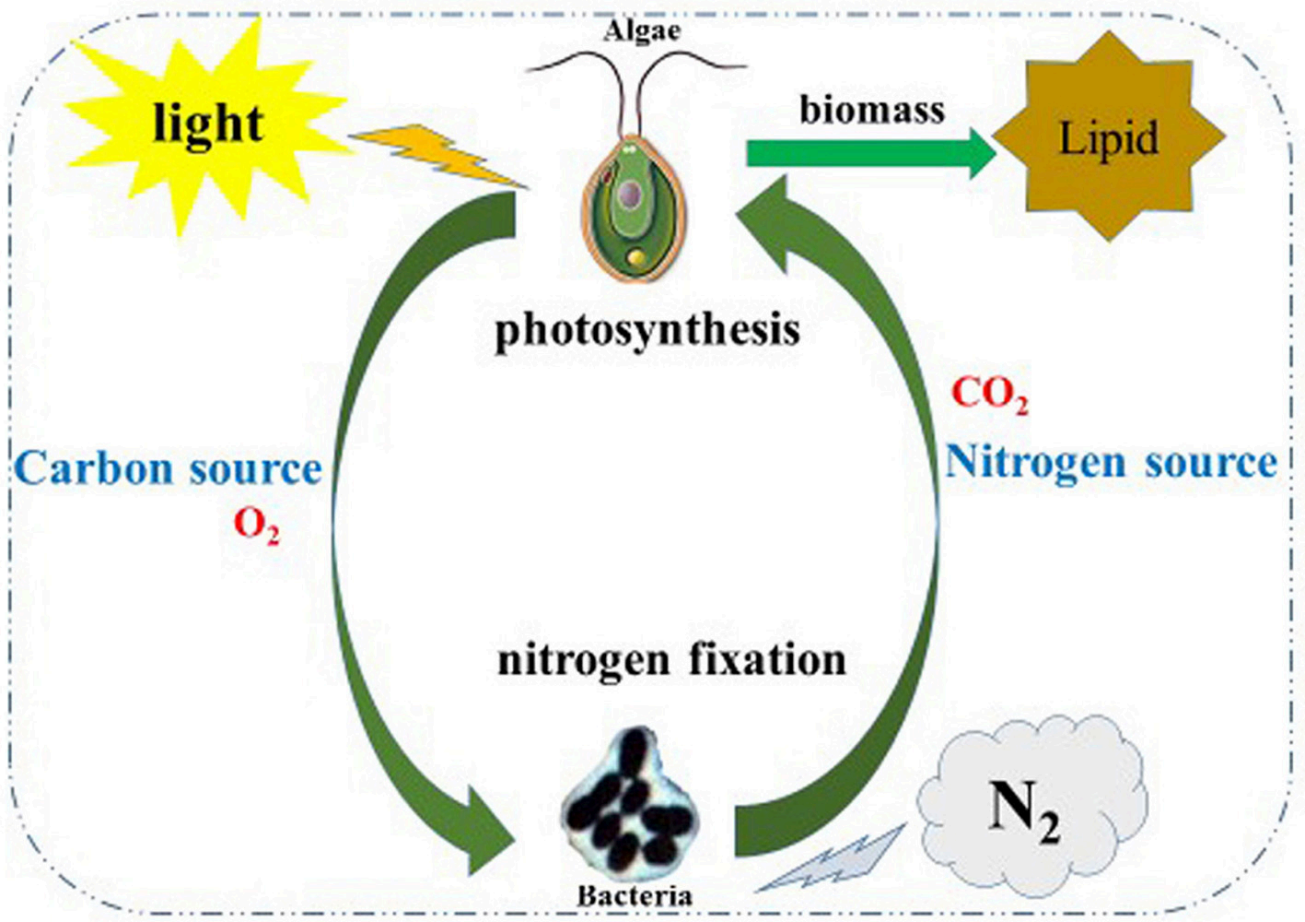
Schematic illustration of the co-cultivation of the algae-bacterial system to improve biomass of and lipid production by algae. In this co-system, algae and bacteria could enhance the growth and biomass of each other through material exchange; algae supply carbohydrates and O_2_ by photosynthesis meanwhile *A. chroococcum* supply the nitrogen source and CO_2_ to algae by nitrogen fixation.

Nitrogen is essential to the growth of micro-algae; thus, the growth and biomass of micro-algae are commonly repressed (Fan et al., 2014). In this study, the growth and biomass of *C. reinhardtii* exhibited little inhibition after 3 days, while the growth and biomass of *C. reinhardtii* co-cultured with *A. chroococcum* increased sharply, this resulted in the nitrogen reservoir could sustain the cell growth for a few days. This suggests that the nitrogen reservoir could sustain cell growth for a few days; thus, there were no differences in the growth on the first 3 days of nitrogen deficiency (Fan et al., 2014). With the consumption of stored nitrogen, the growth and biomass of *C. reinhardtii* were repressed. Conversely, the growth and biomass of *C. reinhardtii* co-cultured with *A. chroococcum* increased significantly (Figures 1, 3). *A. chroococcum* is a type of nitrogen-fixing aerobic bacteria that can draw nitrogen from the air (Walker and Yater, 1978); therefore, the nitrogen in the medium is not needed. In our research, the growth of pure *A. chroococcum* both in TAP and TAP-N media was similar, while the growth of *A. chroococcum* increased after it was co-cultured with algae in both media (Figure 2). This result indicated that nitrogen is not a limiting factor for the growth of *A. chroococcum*. Algae and bacteria exhibit mutually beneficial complex symbiotic relationships. Bacteria release a lot of extracellular metabolites, such as amino acids, enzymes, vitamins, carbohydrates, and lipids into the surrounding environment, which promote the growth of algae. Algal and bacterial growth is also promoted through metabolic regulations and materials exchange. The growth of *S. obliquus* and *Chlorella* increased by their exposure to *P. diminuta* and *P. vesicularis* (Ietswaart et al., 1994); the growth of *Pseudomonas* cultivated alongside *Skeletonema costatum* was more pronounced than that of *Pseudomonas* cultivated alone (Bell et al., 1974); *Pseudomonas* could secrete glycoprotein to *A. glacialis*, which led to their further growth (Riquelm et al., 1988). In our previous work, *Bradyrhizobium japonicum* improved the biomass and hydrogen production of *C. reinhardtii* (Wu et al., 2012; Xu et al., 2016). Similarly, microalgae were always observed near the surface of one type of nitrogen-fixing bacteria by Gyurjan et al. (1984); during the process, algae and bacteria underwent exosymbiotic action by complementary metabolism. In our research, *A. chroococcum* gathered around *C. reinhardtii* and formed a specific “algae-bacteria aggregate” observable under a microscope (Figure S1). In this composite system, algae and bacteria could enhance the growth and biomass of each other through materials exchange; algae supplied carbohydrates and O_2_ by photosynthesis, while *A. chroococcum* could supply nitrogen and CO_2_ to algae by nitrogen fixation (Gyurjan et al., 1984) (Figure 4).

### Total lipid content, lipid production, and lipid productivity of algae co-cultured with *A. chroococcum*

**Figure 5:**
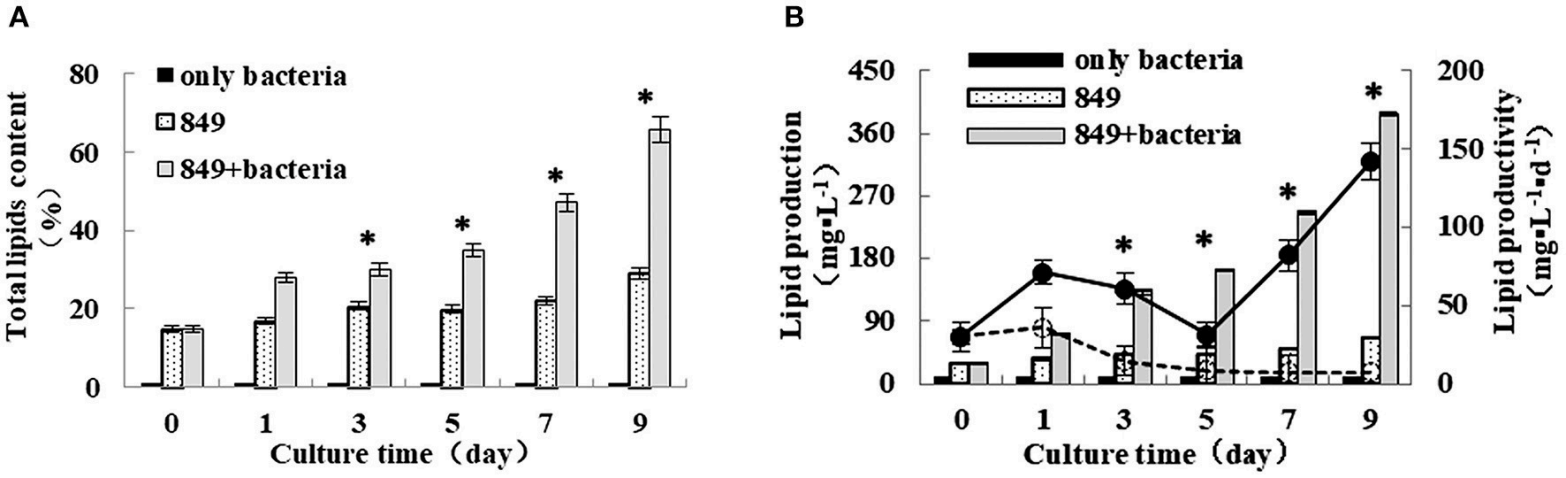
**(A)** Lipid content **(B)** lipid production and lipid productivity of algae in the co-culture were measured on days 0, 1, 3, 5, 7, and 9 after incubation in a nitrogen-deprived medium. Pure algae culture was used as the controls. Lipid content and lipid production are shown with bars and lipid productivity of algae in the co-culture are shown with solid and dashed lines, respectively. ^*^significant difference between the experimental and control groups. The vertical bars indicate standard errors calculated from at least three independent experiments.

To analyze the effect of *A. chroococcum* on lipid production of *C. reinhardtii* in the nitrogen-deficient medium, the algal cells were pre-cultured to saturation and transferred into the nitrogen-deficient medium containing *A. chroococcum* (OD_600_ = 1.0). The pure algal culture served as the control. Samples were extracted at specific points in time to assess the lipid content, lipid production, and lipid productivity. Nitrogen starvation enhances lipid accumulation in microalgae. Consistent with the findings of previous studies, lipid content, lipid production, and lipid productivity of pure algae increased gradually after nitrogen starvation, and those of algae co-cultured with *A. chroococcum* increased profoundly (Figure 5). The lipid content of *A. chroococcum* was not monitored. The maximum lipid content of algae co-cultured with *A. chroococcum* increased from the initial 28.00% to a maximum of 65.85% on day 9, which was 2.3 times that of the pure algae, 29.11% (Figure 5A). Correspondingly, the maximum lipid production and productivity of algae co-cultured with *A. chroococcum* were 387.76 mg/L and 141.86 mg/(L·day), which were 5.9 and 19.4 times the control values, (65.99 mg/(L·day) and 7.33 mg/L, respectively) on day 9 (Figure 5B). A significant difference between lipid production of algae co-cultured with *A. chroococcum* and pure algal culture was observed by the *t-test* on days 3, 5, 7, and 9 (*p* < 0.05). Additionally, algal cells were examined by fluorescent microscopy after staining with the lipid fluorophore Nile Red (Chen et al., 2009). As indicated by Nile Red fluorescence, lipid granules of algae in the co-culture and the lipid body were larger and more numerous than those of pure algae in the nitrogen-rich medium on days 1, 3, 5, 7 and 9 (Figure S2).

*C. reinhardtii* can accumulate lipid even in the absence of certain nutrients, such as nitrogen (Hu et al., 2008; Yeesang and Cheirsilp, 2011; Fan et al., 2014; Park et al., 2015). Therefore, in this study, samples were transferred to a nitrogen-deficient medium when cultured to saturation to induce the lipid accumulation. However, nutrient limitation stimulates lipid accumulation but does so at the expense of growth (Rodolfi et al., 2009; Li et al., 2011). Biomass productivity and lipid content are inversely correlated (Hu et al., 2008; Rodolfi et al., 2009). Similarly, in our study, the biomass of pure *C. reinhardtii* was lower in the nitrogen-deficient medium, but it increased significantly after it was co-cultured with *A. chroococcum* (Figure 3). Consistent with the increased growth and biomass of *C. reinhardtii*, the lipid content, lipid production, and lipid productivity of *C. reinhardtii* in the co-cultures also increased. The increased growth and biomass of *C. reinhardtii* co-cultured with *A. chroococcum* causes the increased lipid content, lipid production, and lipid productivity of *C. reinhardtii* in the co-culture. Because of the number of bacteria added to the algal culture was mostly small, total biomass of pure bacteria slightly increased from the initial value of 0.006 mg to the maximum value of 0.010 mg (Figure 3), the lipid content of *A. chroococcum* was not monitored.

### Fatty acid analysis of algae and *A. chroococcum*

To evaluate the quality and suppliers of lipids from the mixture, the fatty acid profile of lipids in the mixture was analyzed. Pure algae and pure bacteria were used as controls. The FAME composition of algae co-cultured with bacteria and the controls (pure algae and pure bacteria) were determined by GC-MS (Table 2). The fatty acid carbon chain composition of the samples ranged from C8 to C20. The saturated fatty acid in algae, bacteria, and the mixture were C16:0, C18:0, and C19:0. The contents of C16:0 in algae and the mixture were 12.79 and 12.89%, respectively, while C16:0 of bacteria was only 8.15%. The most abundant fatty acid in bacteria was C18:0 and its content was 47.16%, which was 2.39 and 21.05 times its content in algae and bacteria. The contents of C19:0 in algae and the mixture were similar, 3.67% and 4.57%, respectively. The C19:0 content in bacteria was higher than that of them, with a value of 10.97%. The unsaturated fatty acid in algae, bacteria, and the mixture were polyunsaturated fatty acids, mainly C18:2, C18:3. The C18:2 content in algae, bacteria, and the mixture was very low, only the polyunsaturated fatty acids C18:2 in algae and the mixture could be monitored; their values were 2.24% and 1.28%, the polyunsaturated fatty acids C18:2 were not detected in bacteria. Interestingly, C18:3 of algae was 49.53%, which was almost half the ratio of total fatty acids. Inversely, there was almost no C18:3 in bacteria (0.01%) and the amount of the C18:3 of the mixture was 17.43%.

**Table 2:** Fatty acid analysis of algae co-cultured with bacteria, pure algae, and pure bacteria.

**Fatty acid composition**		**Content (%)**		
		***C. reinhardtii***	***A. chroococcum***	***C. reinhardtii* + *A. chroococcum***
Saturated fatty acids (SFA)	C8:0	0.01	0.01	0.07
	C10:0	0.02	0.14	0.23
	C11:0	0.00	0.00	0.01
	C12:0	0.09	7.88	1.57
	C13:0	0.01	0.01	0.01
	C14:0	2.00	12.43	4.00
	C15:0	0.42	0.11	0.23
	C16:0	12.79	8.15	12.89
	C17:0	1.22	0.26	0.97
	C18:0	19.72	2.24	47.16
	C19:0	3.67	10.97	4.57
	C20:0	0.57	0.01	2.35
	C21:0	0.01	0.01	0.02
	C22:0	0.08	0.00	0.56
	C23:0	0.00	0.00	0.01
	C24:0	0.09	0.00	0.30
Monounsaturated fatty acids (MUFA)	C14:1	0.02	0.11	0.06
	C15:1	0.18	0.00	0.09
	C16:1	1.40	38.20	3.94
	C17:1	0.18	0.49	0.10
	C20:1	0.19	0.01	0.14
Polyunsaturated fatty acids (PUFA)	C18:2	2.24	0.00	1.28
	C18:3	49.65	0.01	17.47
	C20:2	0.02	0.01	0.02
	C20:3	0.15	0.00	0.04
	C20:4	0.01	0.00	0.02

The results of the fatty acid analysis indicated that the composition and content of fatty acids in algae, bacteria, and the mixture were different. The highest contents of fatty acids in the three types of samples were C18:3, C16:1, and C18:0, respectively. In our study, the minimum and maximum values of bacterial biomass in the co-culture were 0.002 mg and 0.010 mg (Figure 3); therefore, the proportion of bacteria in the mixture was low and the lipid yield in the mixture was mainly supported from *C. reinhardtii*. Nonetheless, the contents of C19:0 in the mixture was 4.57%. As we all know, algae do not produce any odd number fatty aicds in general, therefore, all the odd number fatty aicds in mixture may supported by the biomass of bacteria. The change in the fatty acid content of the mixture is not caused by the contribution of fatty acids in bacteria but by metabolic regulations and materials exchange between algae and bacteria (Gyurjan et al., 1984) (Figure 4 and Figure S1).

### Effect of *A. chroococcum* on cellular biochemical components of *C. reinhardtii* under nitrogen-deprived conditions

**Figure 6:**
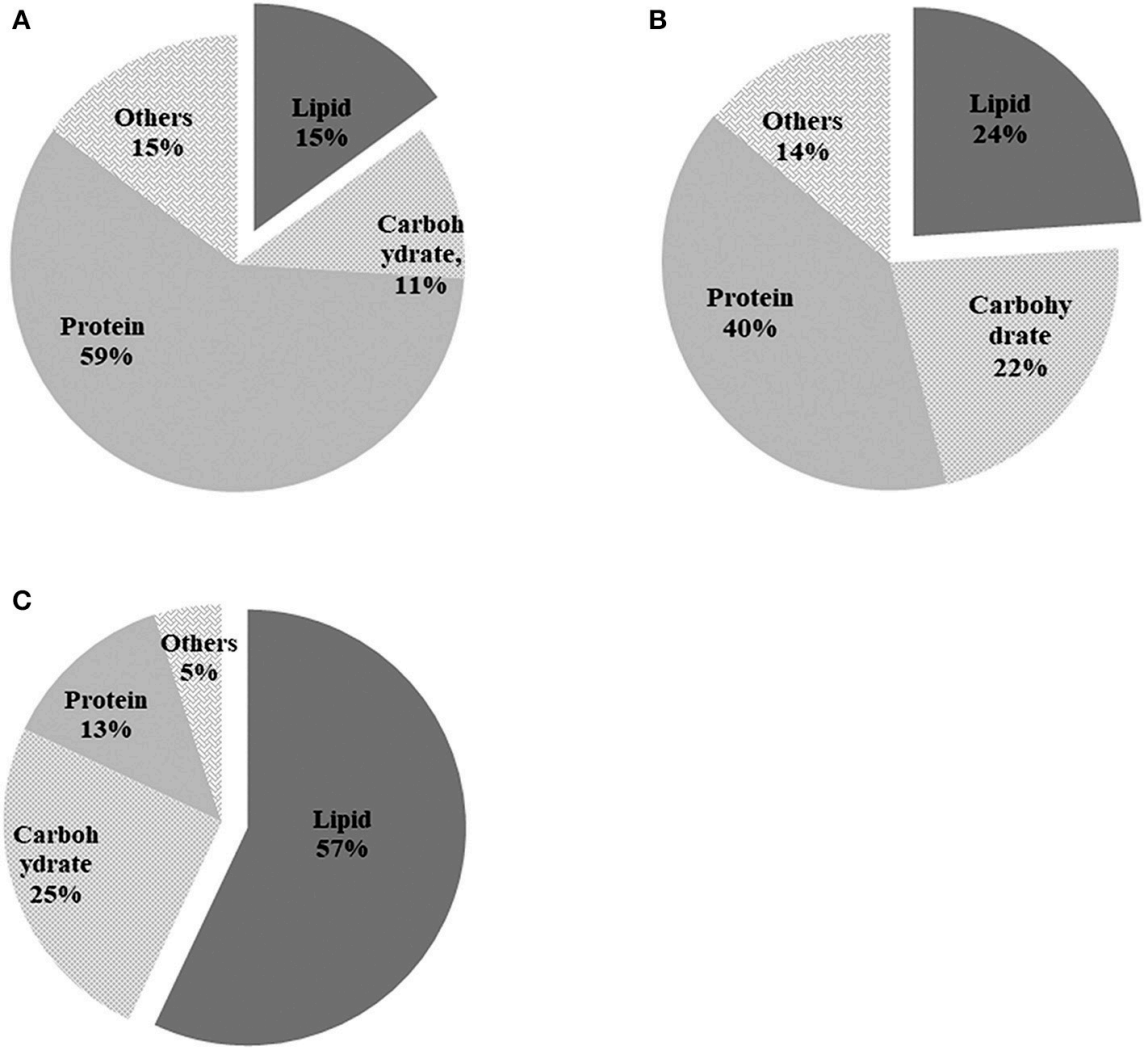
Biochemical composition of pure algae in **(A)** TAP medium before incubation in the nitrogen-deprived medium; **(B)** pure algae; and **(C)** algae in the co-culture after 9 d of incubation in the TAP-N medium. Pure algae in the TAP medium and pure algae incubated for 9 d in the TAP-N medium were used as controls. Light intensity was 200 μE·m^−2^·s^−1^ and the volume ratio of bacteria (OD_600_ = 1.0) and (OD_750_ = 1.0) was 1:40. All the data are the means of three independent experiments with triplicates performed for each experiment.

The components of algal cells were analyzed after nitrogen depletion and the results are shown in Figure 6. Total cellular composition analysis indicated that the pure algal cells consisted of 15% lipids, 11% carbohydrate, and 59% protein in the TAP medium on day 0 (Figure 6A). Nitrogen depletion caused the lipid content of pure *C. reinhardtii* to increase from 15 to 24% (Figure 6B), while the lipid content of *C. reinhardtii* in the co-culture increased significantly, peaking at 57% on day 9 (Figure 6C), which was 2.2 times that of pure *C. reinhardtii*. Inversely, the protein content of pure *C. reinhardtii* decreased from an initial value of 59% to 40% (Figure 6B), and the protein content of *C. reinhardtii* in the co-culture decreased to 13% on day 9, a change of 4.5 folds (Figure 6C). The carbohydrate content in pure *C. reinhardtii* increased from the initial value of 11–22% (Figure 6B)and the lipid content of *C. reinhardtii* in the co-culture increased to 25% (Figure 6C), which was 1.1 times that in pure *C. reinhardtii*. In summary, the lipid and carbohydrate content of pure algae and algae in the co-culture gradually increased while the protein content decreased after the onset of nitrogen deficiency; thus, it is likely that nitrogen deficiency causes the protein in algae to transform into lipid or carbohydrates. After the addition of *A. chroococcum*, the lipid and carbohydrate content in algae became much higher than in pure algae, while the protein content was lower than that of in pure algae. These changes were pronounced on day 9. This indicated that *A. chroococcum* facilitated the transformation of proteins in algae to lipids or carbohydrates. Nitrogen deficiency can cause significant increase in the lipid content of microalgae and a drop in protein content. Ho et al. (2014) found that nitrogen deficiency could dramatically increase the lipid content in algae but has little effect on carbohydrate accumulation, similar to our results. Yen et al. (2013) and Siaut et al. (2011) also found that *C. reinhardtii* could transform protein or peptides to lipids or carbohydrates.

### Expression of lipid biosynthesis genes in *C. reinhardtii* co-cultured with *A. chroococcum* under nitrogen-deprived conditions

**Figure 7:**
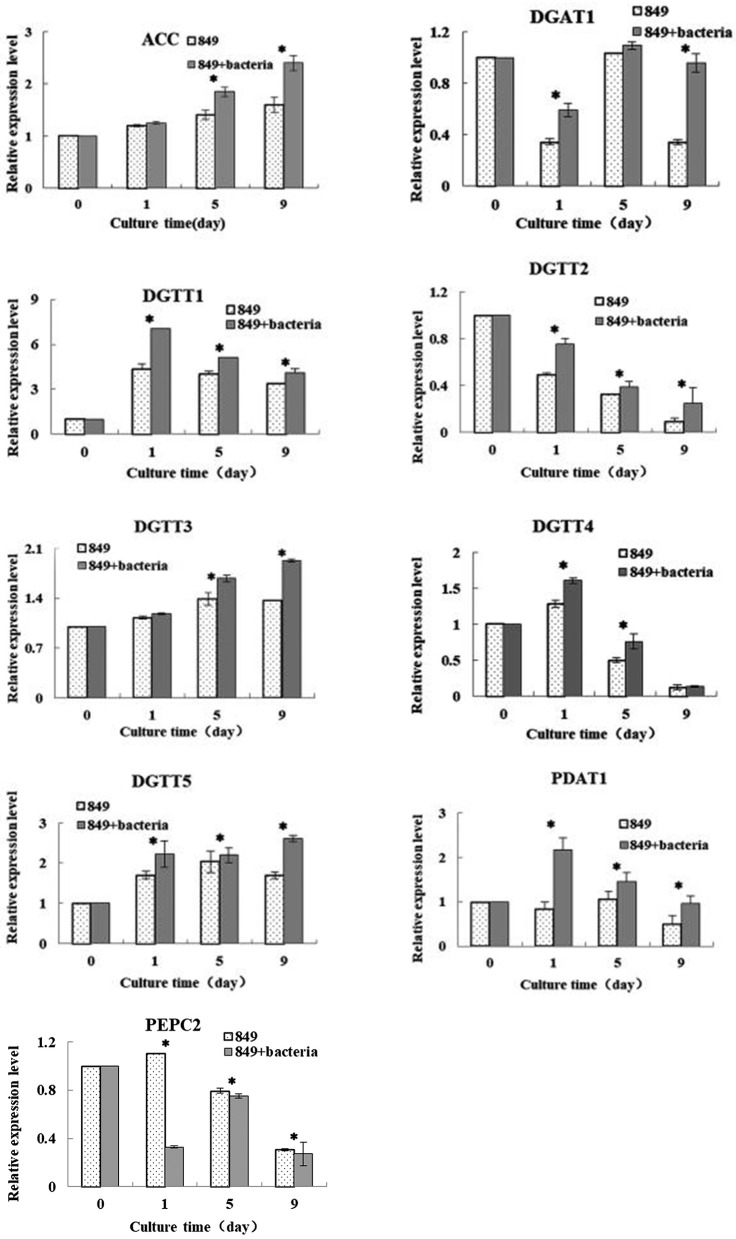
Expression levels of lipid synthesis genes of C. *reinhardtii* in the co-culture on day 0 (in TAP medium), 1 (the initial stage of lipid production), 5 (logarithmic growth period of lipid production), and 9 (the stable growth period of lipid production) in the TAP-N medium. Examined genes included those encoding diacylglycerol: acetal-CoA carboxylase (ACCase); acyl-CoA aceyltransferases type 1 (DGAT1); and type 2 (DGTT1, DGTT2, DGTT3, and DGTT4); phosphoenolpyruvate carboxylase (PEPC); Phospholipid: diacylglycerol acyitransferase (PDAT). Algae cells were used as controls. The vertical bars indicate standard errors calculated from at least three independent experiments.

To explore the reasons for the increased lipid production by *C. reinhardtii* upon addition of *A. chroococcum*, we compared the transcription levels of key genes that dominate the lipid metabolism on day 0 (in TAP medium), day 1 (starting point), day 5 (exponential growth phase), and day 9 (stationary phase) (Figure 7). The results were assessed using Spearman correlation analysis (using SPSS 19.0) to determine the quantitative relationship between the expression level of these genes and lipid content under nutrient-deficient conditions. The results provide an overall perspective for the mechanisms of improved lipid accumulation in response to nutrient stress. The *actin* gene from *C. reinhardtii* was used as an internal control and three biological repetitions were completed; the average CT value of *actin* was 20.3.

Acetyl-CoA carboxylase (ACCase) is a key rate-limiting enzyme that catalyzes the first step in the synthesis of fatty acids and plays an important role in fatty-acid biosynthetic synthesis and catabolism (Cronan and Waldrop, 2002). The levels of expression of the *ACC* gene in *C. reinhardtii* in the co-culture and in the control were analyzed. The results indicated that levels of expression of both *C. reinhardtii* in the co-culture and pure *C. reinhardtii* increased with the increased lipid production and the expression of *ACC* in *C. reinhardtii* in the co-culture was higher than that of the control. A significant difference between the levels of expression of the *ACC* gene in algae co-cultured with *A. chroococcum* and in pure algal culture was observed by the *t-test* on days 5 and 9 (*p* < 0.05). Adding *A. chroococcum* into the algal culture led to a significant increase in the nitrogen deprived medium relative to the control values and the peak level of expression of *C. reinhardtii* in the co-culture was 1.5 times control levels. The lipid content, lipid production, and lipid productivity peaked on day 9 (Figure 5). Carbon from fatty acids is made available from the pool of acetyl-Coenzyme A (CoA) present in the plastid and acts as a precursor for the fatty acid synthesis pathway. An ACCase can catalyze the first reaction of the fatty acid biosynthetic pathway and transform acetyl CoA and CO_2_ into malonyl CoA (Hu et al., 2008). The pattern of ACCase in fatty acid biosynthesis has been thoroughly investigated and it has been proposed that increased ACCase activity is an effective method of stimulating the accumulation of lipids in *Chlorella* (Hsieh and Wu, 2009). Another study showed that lipid accumulation increased with the increased ACCase activity of *Chlorella sorokiniana* (Wan et al., 2011). Similarly, Fan et al. (Fan et al., 2014) reported that the ACCase activity of *Chlorella pyrenoidosa* exhibited a large increase when it was subjected to nitrogen starvation. The role of the ACCase gene expression in *Crypthecodinium cohnii* was studied by Liu et al. and the results indicated that the growth and lipid accumulation were higher in a *C. cohnii* mutant with high ACCase activity (Liu et al., 2017). In our study, the levels of expression in both *C. reinhardtii* in the co-culture and pure *C. reinhardtii* were higher than those in pure *C. reinhardtii* cultured in a nutrient-rich medium. The levels of expression in *C. reinhardtii* co-cultured with *A. chroococcum* were higher than those in the controls. Our results were consistent with those of previous studies.

Diacylglycerol acyltransferase (DGAT) catalyzes the biosynthesis of triacylglycerol (TAG) by the reaction of diacylglycerol with acyl-CoA, and it is the only catalyzing enzyme in the Kennedy pathway that participates solely in the biosynthesis of TAG. The enzyme is an important regulator of lipid biosynthesis, involved in lipid metabolism and lipid deposition. There are two isoforms of DGAT, of which DGAT1 acts extensively on the metabolism of triglycerides and DGAT2 on the accumulation of TAG under nitrogen-limited conditions. In *C. reinhardtii*, the DGAT2 are encoded by five genes in *C. reinhardtii, DATT1, DATT2, DATT3, DATT4, DATT5* (Sugimoto et al., 1989; Miller et al., 2010). The levels of expression of each gene of *C. reinhardtii* in the co-culture and pure algae were assessed under nitrogen deficient conditions. The results showed large differences between the sample in the co-culture and the control. The results of the real-time quantitative PCR indicated that the level of expression of *DGAT1* in *C. reinhardtii* in the co-culture and pure algae decreased during day 1, then increased through day 5. Addition of *A. chroococcum* led to an increase of the expression level of *DGAT1* on day 9. The expression level of *DGTT1* increased dramatically on day 1, then gradually decreased from day 1 through day 9 and reached a minimum on day 9. The levels of expression levels of *DGTT2* and *DGTT4* in *C. reinhardtii* in the co-culture and pure algae decreased from day 1 to day 9 and reached a minimum on day 9, while those of *C. reinhardtii* in the co-culture were higher than those of the control, (1.5 and 1.3 times the control levels, respectively) on day 1. Inversely, the levels of expression of *DGTT3* and *DGTT5* in both co-cultured *C. reinhardtii* increased from day 1 to day 9, peaking on day 9. The levels of expression of all six DGAT genes in *C. reinhardtii* in the co-culture were higher than those of the controls. It is likely that adding *A. chroococcum* led to an increase in the level of expression of DGAT genes. A significant difference between the levels of expression of the *DGAT1* gene in algae co-cultured with *A. chroococcum* and pure algal culture was observed by *t-test* on days 1 and 9 (*p* < 0.05); the levels of expression of the *DGTT1, DGTT2*, and *DGTT5* genes showed significant differences on days 1, 5, and 9 (p < 0.05); the levels of expression of the *DGTT3* gene showed significant difference on days 5 and 9 (*p* < 0.05); and the levels of expression of the *DGTT4* gene showed significant difference on days 1 and 5 (*p* < 0.05). Miller et al. (2010) investigated the transcriptomic analysis of photoheterotrophic *C. reinhardtii* under nitrogen-deprived conditions. In their research, *DGTT1* showed a significant increase and the expression of other *DGAT* genes changed little or not at all. In a study by Msanne et al. (Msanne et al., 2012), *DGTT1* and *DGTT3* displayed a large increase as a response to nitrogen starvation. Unlike in the current work, *DGTT4* was also expressed at a high level. Adding *A. chroococcum* to the algal culture resulted in a large increase in the expression levels of *DGAT1, DATT1, DATT2, DATT3, DATT4*, and *DATT5*, which suggests that the increased expression level of DGAT genes may contribute to lipid synthesis of *C. reinhardtii* co-cultured with *A. chroococcum* subjected to nitrogen starvation. This is another important reason for the increased lipid accumulation of *C. reinhardtii* after addition of *A. chroococcum* to the culture.

The enzyme phosphoenolpyruvate carboxylase (PEPC) is involved in the regulation of photosynthesis and photorespiration. It is also involved in the replenishment of amino acid metabolism. It catalyzes the formation of oxaloacetate into pyruvate and then enters the protein metabolism pathway. As shown in the results of *PEPC2* gene expression, we also detected gene encoding in *C. reinhardtii, PEPC2*. In our work, the levels of expression of *PEPC2* were determined under nutrient-deficient conditions. The results showed that the level of expression of *C. reinhardtii* co-cultured with *A. chroococcum* and pure algae decreased from day 1 to day 9 under nutrient-deficient conditions. The levels of the genes expression of *C. reinhardtii* co-cultured with *A. chroococcum* declined much faster than those of the pure algae. The levels of expression of the *PEPC* gene showed significant differences on days 1, 5, and 9 (*p* < 0.05). This demonstrated that nitrogen depletion leads to the inhibition of the expression of *PEPC2*. After the addition of *A. chroococcum*, the expression of *PEPC2* was more severely inhibited. In theory, when *PEPC* expression is inhibited, more pyruvate will become acetyl-CoA through a process catalyzed by pyruvate dehydrogenase, which facilitates lipid synthesis. Nitrogen as a signal molecule can be induced and regulated by the expression of genes. *PEPC* gene expression, protein content and *PEPC* gene activity were highly positive (Sugimoto et al., 1989; Chen et al., 1998). Therefore, *PEPC* plays a negative regulatory role in lipid production. Blocking the *PEPC* gene can increase lipid accumulation in many species (Sugimoto et al., 1989; Chen et al., 1998). Lipid content increases dramatically with decreased expression of the *PEPC2* gene in *C. reinhardtii* under nitrogen-deficient conditions (Deng et al., 2011). This is consistent with our results.

Phospholipid diacylglycerol acyltransferase (PDAT) is an acyl-CoA independent enzyme that transfers the acyl group from the sn-2 position of a phospholipid to the sn-3 position of a diacylglycerol (Boyle et al., 2012). Boyle et al. (2012) reported lipid accumulation by a *C. reinhardtii* mutant lacking the *PDAT1* gene at 25% of wild algae, indicating that *PDAT1* plays an important role in the lipid synthesis of *C. reinhardtii*. In our research, the levels of expression of both *C. reinhardtii* in the co-culture and pure *C. reinhardtii* increased with the increased lipid production. The expression levels of *C. reinhardtii* in the co-culture were higher than those of the control; i.e., adding *A. chroococcum* to the algal culture led to a significant increase in the expression level of the *PDAT1* gene in the depletion of the nitrogen medium. The levels of expression of the *PDAT1* gene showed significant differences on days 1, 5 and 9 (*p* < 0.05). Especially, the lowest level of expression of the *PDAT1* gene in *C. reinhardtii* in the co-culture was 1.5 times the control levels on day 9, which is when the lipid content, lipid production, and lipid productivity were the greatest (Figure 3).

## Conclusions

In this study, we co-cultured *C. reinhardtii* with *A. chroococcum* to enhance lipid accumulation of *C. reinhardtii* by increasing the growth and biomass of *C. reinhardtii* under nitrogen-deprived conditions. After the addition of *A. chroococcum*, the growth and biomass of *C. reinhardtii* increased, as well as lipid accumulation, lipid content, and lipid productivity. In summary, *A. chroococcum* improved the lipid accumulation and the activity of *C. reinhardtii* by enhancing the growth, biomass, and levels of expression of genes that positively regulate lipid metabolism and by decreasing the expression levels of genes that negatively regulate lipid metabolism. This study provides an effective method for increasing the lipid production of *C. reinhardtii* by increasing its biomass and through its ecological relationship with *A. chroococcum*.

## Author contributions

LX and QW proposed the idea and hypothesis. XC carried out the experiment. LX and XC analyzed data and drafted the manuscript. All authors read and approved the final manuscript for publication.

### Conflict of interest statement

The authors declare that the research was conducted in the absence of any commercial or financial relationships that could be construed as a potential conflict of interest.
