# Supplementary figures and images for: Enhanced Lipid Production in Chlamydomonas reinhardtii by Co-culturing With Azotobacter chroococcum
Source: Front Plant Sci. 2018 Jun 28;9:741. doi: 10.3389/fpls.2018.00741 (PMC6032324; doi:10.3389/fpls.2018.00741)

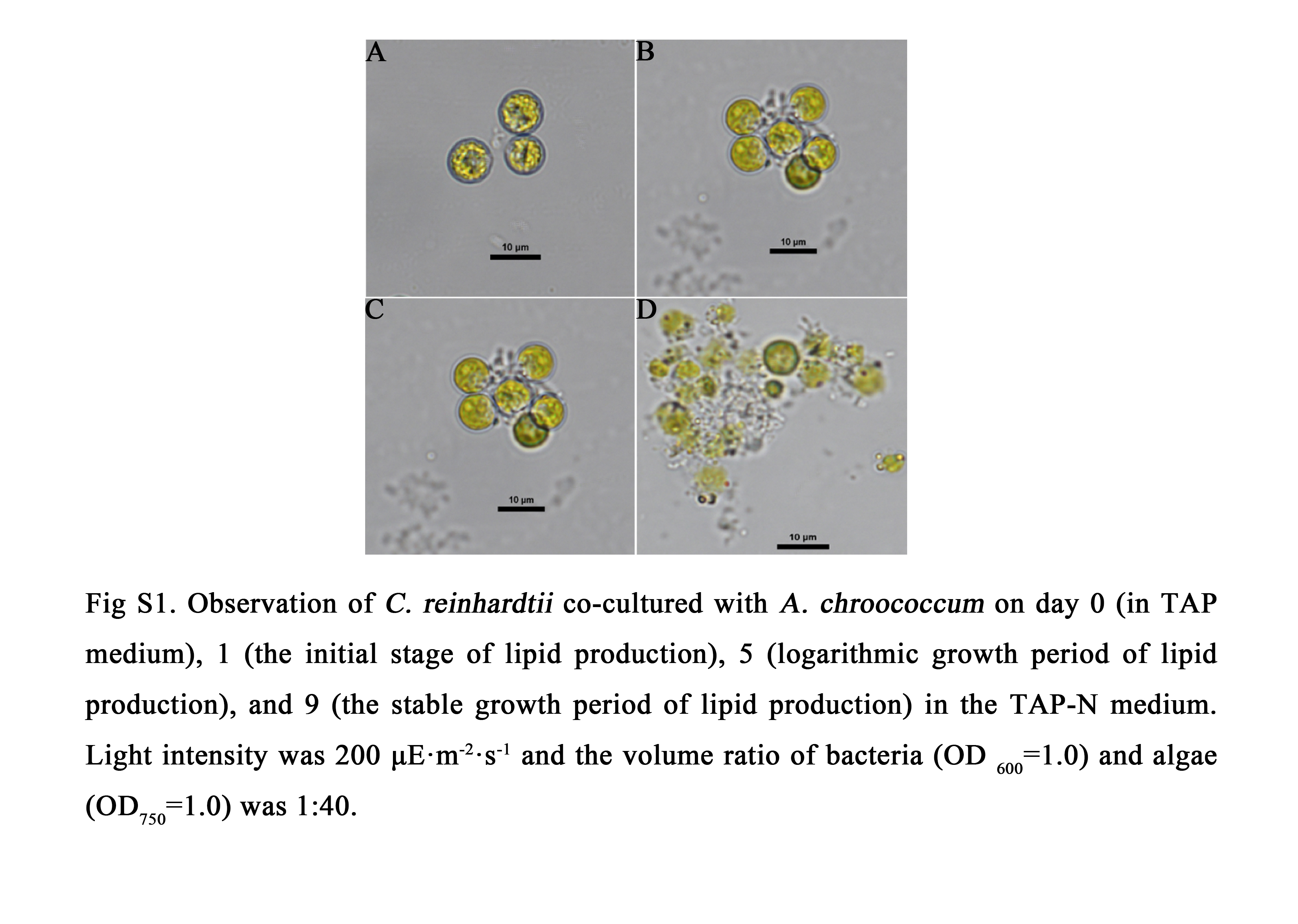

Supplement: Supplementary file 1 [file Image_1.JPEG]

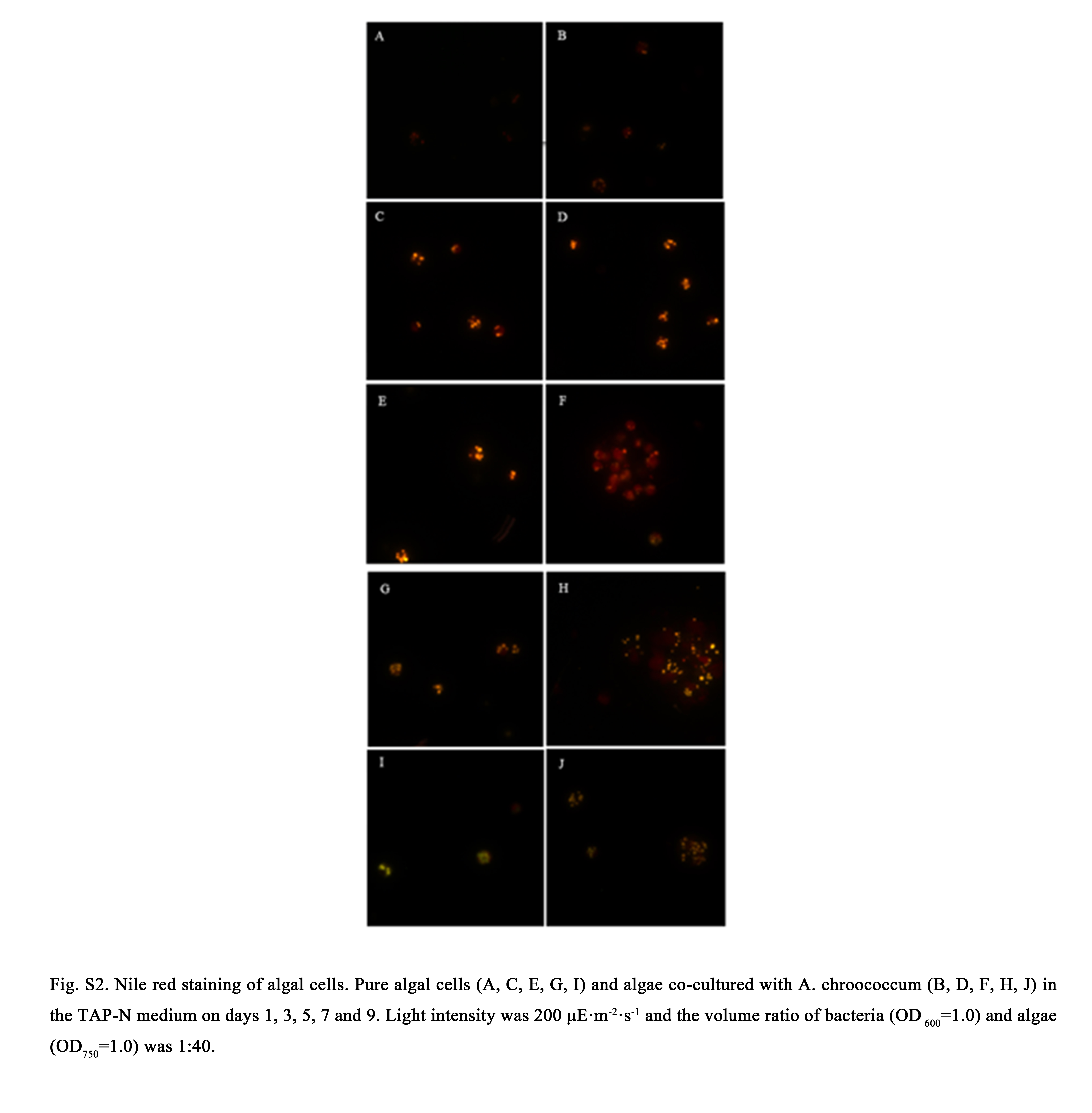

Supplement: Supplementary file 2 [file Image_2.JPEG]
